# Prevalence, Phenotype, and Correlates of Avoidant/Restrictive Food Intake Disorder Symptoms in the Gulf Cooperation Council: An Underserved Region

**DOI:** 10.1002/eat.24400

**Published:** 2025-03-14

**Authors:** Bernou Melisse, Hassan Fakhri, Lynne Kennedy, Maria J. Figueiras, Munirah Alshebali, Hala Abu Taha, Carine el Khazen, Dalal Alkazemi, Sandra Mulkens

**Affiliations:** ^1^ American Center for Psychiatry and Neurology Abu Dhabi Abu Dhabi UAE; ^2^ Co‐Eur Utrecht the Netherlands; ^3^ Department of Clinical Psychology Utrecht University Utrecht the Netherlands; ^4^ Department of Medical and Clinical Psychology Tilburg University Tilburg the Netherlands; ^5^ Department of Public Health Qatar University Doha Qatar; ^6^ College of Natural and Health Sciences, Department of Psychology Zayed University Abu Dhabi UAE; ^7^ Technical Sciences Programs Princess Nourah Bint Abdulrahman University Riyadh Saudi Arabia; ^8^ College of Life Sciences, Department of Food Science and Nutrition Kuwait University Al‐Shadadiya Kuwait; ^9^ Department of Clinical Psychological Science Maastricht University Maastricht the Netherlands

**Keywords:** avoidant/restrictive food intake disorder, clinical presentation, eating disorders, Gulf cooperation council, phenotype, prevalence

## Abstract

**Introduction:**

Prevalence estimates and correlates of ARFID in non‐Western samples are lacking. This study aims to estimate the prevalence of ARFID symptoms, identify its phenotypes, and explore its correlates in a community sample from the Gulf Cooperation Council (GCC).

**Method:**

Participants were parents of children aged 4–13 years (*n* = 87) and individuals of ≥ 14 years old (*n* = 433). They completed the Pica, ARFID, and Rumination Disorder Interview‐ARFID‐Questionnaire (PARDI‐AR‐Q), the Nine Item ARFID Screen (NIAS) and the Eating Disorder Examination‐Questionnaire (EDE‐Q). Multiple regression analyses were performed with body mass index or its standard deviation score, comorbid psychopathology, EDE‐Q global score, sex, and age as independent variables; the dependent variable was ARFID psychopathology.

**Results:**

Among individuals not reporting eating disorder symptoms driven by overvaluation of shape and weight, the PARDI‐AR‐Q diagnostic prediction suggested that approximately 23.4% of those aged ≥ 14 exhibited ARFID symptoms. Based on the NIAS, sensory‐based food avoidance was the most reported phenotype expression, with approximately 29.4% of children (4–13 years) and 12.8% of adolescents/adults (≥ 14‐years) reporting ARFID symptoms. In adolescents and adults, ARFID psychopathology was positively associated with eating disorder pathology driven by overvaluation of shape and weight, with female sex and negatively associated with age.

**Discussion:**

This study is the first to identify a subset of individuals in GCC countries with ARFID symptoms across sexes and a broad age range, with sensory sensitivity as the most common symptom.

**Summary**

- In the GCC, based on the PARDI‐AR‐Q diagnostic prediction, it is suggested that 23.4% of individuals aged ≥ 14 exhibited ARFID symptoms.
- Based on the NIAS, sensory‐based food avoidance was the most reported symptom, with 29.4% of children (4–13 years) and 12.8% of adolescents/adults (≥ 14 years) exhibiting ARFID symptoms.
- Among participants aged ≥ 14 years, having other eating disorder psychopathology, being female, and of younger age were factors associated with ARFID psychopathology.

## 1 Introduction

Avoidant/restrictive food intake disorder (ARFID) is characterized by restrictive eating patterns associated with significant physical and/or psychosocial consequences. ARFID is driven by three primary profiles, namely (1) avoidance based on the sensory characteristics of food (such as taste, smell, color, and texture); (2) a lack of interest in food or eating; and (3) avoidance based on concern over aversive consequences of eating (e.g., choking, gastrointestinal discomfort, or vomiting) (APA 2022). Consequently, individuals with ARFID may become malnourished, exhibit weight loss (Strand et al. 2019), or have an increased body mass index (BMI) due to selectively eating energy‐dense products (Fonseca et al. 2024).

ARFID is commonly associated with comorbid psychopathology, including mood disorders (Archibald and Bryant‐Waugh 2023; Cooney et al. 2018; Kambanis et al. 2020), anxiety disorders (Archibald and Bryant‐Waugh 2023; Cooney et al. 2018; Kambanis et al. 2020; Sanchez‐Cerezo et al. 2023), and neurodevelopmental, disruptive, and conduct disorders (Archibald and Bryant‐Waugh 2023; Kambanis et al. 2020; Sanchez‐Cerezo et al. 2023). Moreover, although other eating disorders are an exclusion criterion for ARFID (APA 2022), patients seeking treatment for these conditions often report co‐occurring ARFID pathology (Hay et al. 2017; Williams et al. 2015).

Prior research indicates that ARFID is more common in males (Cooney et al. 2018; Forman et al. 2014; Norris et al. 2014; Wong et al. 2022), and globally, ARFID affects 3%–5% of children (Bertrand et al. 2021; Chen et al. 2019; Herle et al. 2020; Sanchez‐Cerezo et al. 2023) and 1% of adults (Sanchez‐Cerezo et al. 2023). However, these estimates vary widely between children and adolescents (Sanchez‐Cerezo et al. 2023). Since ARFID was only recently included in the DSM (APA 2013), measures available to identify ARFID and its clinical features are scarce (Dinkler and Bryant‐Waugh 2021). Existing studies are primarily concentrated in the West, with limited data from non‐Western countries, including only one study from South‐East Asia (Ning et al. 2022) and two in the Levant region [Lebanon] (Chaaya et al. 2024; Fekih‐Romdhane et al. 2023). Prevalence estimates and correlates of ARFID symptoms in the Gulf Cooperation Council [GCC], comprising Bahrain, Kuwait, Oman, Saudi Arabia, Qatar, and the United Arab Emirates [UAE] (GCCcouncil 2021), remain undocumented.

ARFID presentations, previously referred to as “Selective Eating Disorder,” (Fonseca et al. 2024; Taylor and Emmett 2019), and later as feeding disorder of infancy/early childhood (APA 2000), existed before their formal recognition in the DSM‐5 (APA 2013), highlighting its recent emergence as a distinct diagnosis. This inclusion has helped increase awareness and research into ARFID. Furthermore, in the GCC, economic prosperity driven by the oil‐boom lead to rapid societal and cultural shifts (Melisse et al. 2020). These changes disrupt traditional lifestyles, including dietary habits, with decreased exposure to a variety of foods (Shrimpton and Rokx 2012), and may be contributing to ARFID symptoms (Schatzker 2015). Geopolitical instability and widespread trauma in the region could contribute to an increase in eating disorder symptoms (Molendijk et al. 2017). However, it is critical to emphasize that these factors should not be viewed as the sole contributors to the prevalence of ARFID. Instead, they intersect with the broader context of increased awareness and recognition of the disorder, which has led to more diagnoses and better understanding of its clinical characteristics.

Despite the growing global recognition of ARFID, the GCC remains underrepresented in eating disorder research. Furthermore, in this region, few widely used self‐report instruments are available and used to screen eating disorder symptoms, operationalized as a score above the clinical cutoff on a self‐report measure (Hadati et al. 2024; Melisse et al. 2021; Melisse et al. 2022b). However, these self‐reports target deliberate engagement in restrictive/irregular food patterns as part of the overvaluation of shape and weight, which is an exclusion criterion for ARFID (APA 2022), making them unsuitable for screening and diagnosing ARFID symptoms (Kambanis et al. 2024). This gap underscores the need for ARFID‐specific assessments suitable for use in the GCC and consequently explores their concurrent validity and recognition of its unique regional clinical features.

A review examining the prevalence of eating disorders driven by shape and weight concern in the GCC reports comparable prevalences with the West (Melisse et al. 2024b). Similarly to eating disorder symptoms driven by overvaluation of shape and weight, it is suggested that the prevalence of ARFID symptoms in the GCC is underestimated (Melisse et al. 2021). Additionally, information regarding its' clinical presentation is unknown. Furthermore, existing studies from Western populations indicate that ARFID psychopathology is associated with comorbid psychopathology (Archibald and Bryant‐Waugh 2023; Cooney et al. 2018; Kambanis et al. 2020; Sanchez‐Cerezo et al. 2023), eating disorder psychopathology driven by overvaluation of shape and weight (Hay et al. 2017; Williams et al. 2015), male sex (Cooney et al. 2018; Forman et al. 2014; Norris et al. 2014; Wong et al. 2022), inversely associated with age (Bertrand et al. 2021; Chen et al. 2019; Herle et al. 2020; Sanchez‐Cerezo et al. 2023), and a potential association with BMI (Fonseca et al. 2024; Strand et al. 2019). However, it remains unclear whether these associations hold true in the GCC region. Understanding the correlates of ARFID psychopathology can aid in identifying high‐risk groups for targeted prevention programs (Stice et al. 2007). Consequently, addressing ARFID symptoms in this underrepresented region is critical to informing health policy, enhancing diagnostic accuracy, and improving treatment access (Fekih‐Romdhane et al. 2023; Melisse et al. 2020).

The aim of this study was to explore concurrent validity between the PARDI‐AR‐Q and the NIAS, and to estimate the prevalence of ARFID symptoms, including the three ARFID phenotype expressions. The third aim was to examine if, like in the West, BMI or its standard deviation score, other comorbid psychopathology, eating disorder pathology driven by overvaluation of shape and weight, sex, and age were correlates of ARFID psychopathology. This was conducted in the GCC among a community convenience sample consisting of children aged ≥ 4–13 years and adolescents/adults aged ≥ 14 years excluding participants with eating disorder symptoms driven by overvaluation of shape and weight.

## 2 Method

### 2.1 Participants

#### 2.1.1 Inclusion and Exclusion Criteria

Participants included residents of the GCC (i.e., Bahrain, Kuwait, Oman, Saudi Arabia, Qatar, UAE) (GCCcouncil 2021), literate in English and having web access. Additionally, participants were parents of children aged between 4 and 13 years, or individuals of ≥ 14 years old. In the first case, parents had to be willing to complete the self‐report instruments. Residents of a non‐Gulf country and insufficient proficiency in English were excluded.

### 2.2 Recruitment and Procedures

Data were collected anonymously, using a purposive community‐based convenience sample and snowballing, which were chosen because it is challenging to gather data from GCC citizens, who are less inclined to participate in research (Melisse et al. 2022a). Study participation was invited via online platforms (X, Instagram, Facebook, LinkedIn) and WhatsApp groups in the GCC. No responses were received from Bahrain or Oman. The invitation directed participants towards the web‐based survey, where they could choose to complete the self‐report (i) about their child aged 4–13 years or (ii) about themselves if aged ≥ 14 years informed consent was obtained from parents for children, and both parents and adolescents for ages 14–18 by ticking a box. Participants completed the survey anonymously and were debriefed afterward, with contact details of the first author (BM) for further assistance and information on treatment options. The survey was available in English. Ethical approval was obtained on 11‐07‐2024 from the American Center for Psychiatry and Neurology's ethical board (reference number ACPN_0062). Data collection occurred between July 2024 and September 2024. Data were processed and stored in Qualtrics (Qualtrics 2024).

### 2.3 Measures

#### 2.3.1 Pica, ARFID, and Rumination Disorder Interview‐ARFID‐Questionnaire (PARDI‐AR‐Q)

The PARDI‐AR‐Q (Bryant‐Waugh et al. 2022) is the self‐report version of the Pica, ARFID, and Rumination Disorder Interview (Basel et al. 2019). The PARDI is the most widely used structured clinical interview to diagnose ARFID. Like the PARDI, the PARDI‐AR‐Q includes items aligned with the DSM‐5 diagnostic criteria and a diagnostic algorithm designed to differentiate individuals screening positive or negative for ARFID, operationalized as a diagnostic prediction for ARFID symptoms. In addition, the PARDI‐AR‐Q measures ARFID psychopathology with a severity of impact score, and with three subscale scores based on the three ARFID phenotypes (avoidance based on the sensory characteristics of food, lack of interest in eating or food, and concern about aversive consequences of eating). The questionnaire has 32 items: most are answered on a binary scale (yes/no), and 11 on a 7‐point Likert scale (0–6). Scores are averaged, with higher scores indicating more severe ARFID psychopathology. The PARDI‐AR‐Q severity of impact score is based on the sum score of items 22 and 23 divided by 2, resulting in participants with a score above zero on one of these items having a PARDI‐AR‐Q severity of impact score. Each subscale score is based on three items.

Finally, BMI or BMI standard deviation score, a widely applied statistical measure in pediatric populations, used to evaluate an individual's BMI relative to the mean BMI of a reference population, accounting for age and sex (WHO 2024), was calculated based on weight, height, and date of birth provided in the PARDI‐AR‐Q. Two versions of the questionnaire are available: one for parents or caretakers of children aged 4–13 and the other for participants aged 14+. The PARDI‐AR‐Q showed sufficient convergent, divergent, and concurrent validity(*r* = 0.70–0.70) (Bryant‐Waugh et al. 2022).

#### 2.3.2 Nine‐Item Avoidant/Restrictive Food Intake Disorder Screen (NIAS)

The PARDI‐AR‐Q is preferred as it is the self‐report version of the PARDI, though its validation is limited to adults, adolescents, and a control group in the United States. Therefore, the NIAS was also employed, as it is validated for Arab cultures. The NIAS is a self‐report that measures the symptoms of the three ARFID phenotype expressions. A total of nine items, divided over 3 subscales, are answered on a 6‐point Likert scale (0: strongly disagree, to 5: strongly agree) (Zickgraf and Ellis 2018). Each subscale includes three items based on one of the three ARFID phenotypes: “picky eating”, “fear”, and “low appetite”. Higher scores indicate more severe ARFID psychopathology. Total scores are calculated as the sum of all nine items (range: 0–45), and subscale scores as the sum of the relevant three items (range: 0–15). Proposed cut‐off values indicating ARFID symptoms for the concurrent ARFID phenotype for the subscales of the original NIAS are: picky eating ≥ 10, fear ≥ 9, and low appetite ≥ 10 (Burton Murray et al. 2021). The NIAS was previously examined among Arab populations in Lebanon, showing good psychometric properties, including high internal consistency (McDonald's *w* = 0.75–0.90) and a three‐factor structure (Fekih‐Romdhane et al. 2023).

#### 2.3.3 Eating Disorder Examination Questionnaire (EDE‐Q)

The EDE‐Q is the self‐report version of the Eating Disorder Examination interview (Cooper and Fairburn 1987; Fairburn and Beglin 2008). The EDE‐Q is widely used to assess the severity of eating disorder psychopathology and behaviors, including overvaluation of shape/weight, overeating, and binge eating over the past 28 days (Fairburn and Beglin 2008). The 28 items are answered on a 7‐point Likert scale (0: absent, 6: markedly present/every day). The global score is calculated as the average score of all items. The cut‐off indicating symptoms of an eating disorder driven by shape and weight concerns in the Arabic version is 2.93 (Melisse et al. 2021). The DSM‐5 precludes the diagnosis of an eating disorder driven by overvaluation of shape and weight (APA 2022). Consequently, a score exceeding the clinical cutoff was used to operationalize this DSM‐5 criterion in the present study and, therefore, was considered an exclusion criterion for a positive ARFID screen. The EDE‐Q is suitable for use among Arab populations and has a single‐factor structure and a high internal consistency (Cronbach's *α* = 0.93) (Melisse et al. 2021). Internal consistency of the EDE‐Q was excellent in the present study (4–13 years old: McDonalds *w* = 0.96; ≥ 14‐years old: McDonalds *w* = 0.89).

#### 2.3.4 Demographics

Demographic variables were collected through self‐report, including ethnicity, age, sex, highest level of education attained, and marital status. Participants were also asked to declare if they were diagnosed with any other disorder.

### 2.4 Sample Size

To achieve 80% power in a regression analysis with five predictors and an effect size of 0.15, a sample size of approximately *N* = 91 is required for each age group to detect a medium effect size based on *p* < 0.05.

### 2.5 Statistical Analysis

#### 2.5.1 Software and Psychometric Properties

All analyses were performed in SPSS version 29 and 30 (IBM Corp, 2024). Internal consistencies were measured by McDonald's *ω* (≥ 0.70 acceptable, and ≥ 0.90 excellent) (MacDonald 1999). Concurrent validity was examined by bivariate correlations between the PARDI‐AR‐Q and the NIAS subscale scores (≥ 0.70 acceptable) (Spearman 1961).

#### 2.5.2 Prevalence Estimation of ARFID Symptoms

(Sub)scale scores on the ARFID self‐reports were calculated for individuals who did not exhibit symptoms of an eating disorder driven by the overvaluation of shape and weight (EDE‐Q global‐score < 2.93). In addition, the PARDI‐AR‐Q diagnostic prediction was used to identify ARFID symptoms, and exceeding the NIAS subscale cutoffs was used to identify the presence of the ARFID phenotypes. Independent samples *t*‐tests were used to examine whether subscale scores varied over the age groups.

#### 2.5.3 Correlates

Among individuals not exhibiting symptoms of an eating disorder driven by overvaluation of shape and weight, two multiple regression analyses were conducted for each age group using the stepwise method. The independent variables included BMI (for individuals aged ≥ 14 years) or its standard deviation score (for those aged 4–13 years), psychopathology other than an eating disorder, EDE‐Q global‐score, sex, and age. The dependent variable was ARFID psychopathology, measured using the PARDI‐AR‐Q severity of impact score and the NIAS total score.

## 3 Results

### 3.1 Participants

A total of 520/588 (89.7%; *n* = 87 aged 4–13 years old, *n* = 433 aged ≥ 14 years old) participants completed demographics and all self‐reports. There were no differences between completers and non‐completers in the sample aged 4–13 years old. Participants who completed all questionnaires were higher educated than the non‐completers in the sample aged ≥ 14 years (*t* (491) = 2.6, *p* < 0.001). Table 1 shows the descriptive statistics of both samples.

**TABLE 1:** Demographics of two samples of individuals residing in the Gulf Cooperation Council: One sample of children aged 4–13 years old, and one sample of adolescents and adults aged ≥ 14 years old.

	Participants aged 4–13 years old (*n* = 87)	Participants aged ≥ 14 years old (*n* = 433)
Nationality^a^, *N*, %		
Emirati	28, 32.2%	133, 30.7%
Saudi	20, 23.0%	99, 22.8%
Qatari	0, 0.0%	2, 0.5%
Kuwaiti	3, 3.4%	5, 1.2%
African region (e.g., Mauritania, South Africa)	2, 2.3%	9, 2.1%
Region of the Americas (e.g., Argentina, Mexico)	1, 1.1%	3, 0.7%
South East Asian region (e.g., Bangladesh, Thailand)	3, 3.4%	15, 3.4%
European region (e.g., United Kingdom, Russia)	7, 8%	30, 6.9%
Eastern Mediterranean region (e.g., Bahrain, Egypt)	9, 10.3%	97, 22.4%
Pacific region (e.g., China, Australia)	0, 0.0%	6, 1.4%
Northern America (e.g., United States, Canada)	4, 4.4%	22, 5.1%
Age, mean (SD)	10 (2.9)	29.2 (12.7)
Age cohort, *N*, %		
14–17 years	NA	331, 76.4%
18–66 years	NA	102, 23.6%
Sex, *N*, %		
Female	36, 41.2%	271, 62.6%
Male	51, 58.8%	162, 37.4%
BMI, mean (SD)	21.1 (6.7)	23.7 (7.2)
BMI, standard deviation score	0.4 (0.6)	NA
Marital status, *N*, %		
Married	0, 0.0%	99, 22.7%
Single	87, 100%	209, 48.3%
Divorced	0, 0.0%	15, 3.5%
Engaged	0, 0.0%	5, 1.2%
Other	0, 0.0%	105, 24.2%
Occupation/education, *N*, %		
Nursery	2, 2.3%	0, 0.0%
Elementary school	76, 87.4%	0, 0.0%
High school	9, 10.3%	77, 17.8%
University	0, 0.0%	53, 12.2%
College/vocational education	0, 0.0%	12, 2.8%
Employed	0, 0.0%	140, 32.3%
Unemployed	0, 0.0%	34, 7.9%
Other	0, 0.0%	118, 27.3%
Highest completed level of education, *N*, %		
Kindergarden	20, 23.0%	3, 0. 7%
Elementary school	59, 67.8%	15, 3.5%
High school	8, 9.2%	149, 34.4%
College/vocational education	0, 0.0%	27, 6.2%
University	0, 0.0%	153, 35.3%
Postgraduate for example, doctorate or PhD	0, 0.0%	87, 20.1%
Comorbid psychopathology, *N*, %		
Absent	21, 24.1%	172, 39.7%
Mood disorder, such as major depression	0, 0.0%	62, 14.3%
Anxiety disorder	0, 0.0%	75, 17.3%
Obsessive compulsive disorder	1, 1.1%	20, 4.6%
Autism spectrum disorder	2, 2.3%	1, 0.2%
ADHD	2, 2.3%	32, 7.4%
Post traumatic stress disorder/trauma	2, 2.3%	17, 3.9%
Personality disorder	1, 1.1%	8, 1.9%
Use of medication, *N*, %		
Yes	8, 9.2%	46, 10.6%
No	79, 90.8%	387, 89.4%
Self‐report measures, mean (SD)		
PARDI‐AR‐Q		
Severity of impact	1.2 (1.3)	1.0 (1.5)
Avoidance based on the sensory characteristics of food	1.6 (1.9)	0.8 (1.3)
Lack of interest in eating or food	1.2 (1.6)	1.5 (1.6)
Concern about aversive consequences of eating	0.8 (1.3)	0.8 (1.3)
Diagnostic prediction *N*, %	0, 0.0%	132, 30.3%
NIAS		
Total score	13.1 (8.7)	12.8 (10.0)
Picky eating	7.4 (4.7)	5.0 (4.2)
Fear	2.1 (3.1)	3.5 (3.9)
Low appetite	3.7 (3.7)	4.3 (4.0)
NIAS score above clinical cutoff^b^, *N*, %		
Picky eating	7, 8%	43, 9.9%
Fear	1, 1.1%	36, 8.3%
Low appetite	2, 2.3%	28, 6.4%
EDE‐Q		
Global‐score, mean (SD)	1.0 (1.5)	1.2 (1.3)
Global‐score above clinical cutoff^c^, *N*, %	53, 60.9%	169, 39.0%

Abbreviations: ARFID, avoidant/restrictive food intake disorder; BMI, body mass index; EDE‐Q, Eating Disorder Examination‐Questionnaire; NIAS, nine item avoidant/restrictive food intake disorder screen; PARDI‐AR‐Q, Pica, ARFID and Rumination Disorder Interview—ARFID‐Questionnaire.

^a^ Nationality was based on the WHO regions (WHO 2020).

^b^ NIAS clinical cutoffs: picky eating ≥ 10, fear ≥ 9, and low appetite ≥ 10 (Burton Murray et al. 2021).

^c^ EDE‐Q global‐score clinical cutoff ≥ 2.93 (Melisse et al. 2021).

### 3.2 Psychometric Properties of the PARDI‐AR‐Q and NIAS

Table 2 shows the internal consistencies of the PARDI‐AR‐Q and the NIAS, which were good to excellent in the present study. Addressing the first aim, concurrent validity between the PARDI‐AR‐Q and NIAS subscale scores was supported (*p* < 0.001): sensory‐based avoidance/picky eating: *ρ* = 0.80; *ρ* = 0.60; lack of interest/low appetite: *ρ* = 0.82; *ρ* = 0.60, and concern of aversive consequences/fear: *ρ* = 0.69; *ρ* = 0.56 among the groups aged 4–13 years old and ≥ 14 years, respectively.

**TABLE 2:** Internal consistencies of the PARDI‐AR‐Q and the NIAS.

Self report	Subscale	Age category	McDonald's *ω*
PARDI‐AR‐Q	Avoidance based on sensory characteristics	4–13 years	0.88
		≥ 14 years	0.84
	Lack of interest in eating or food	4–13 years	0.88
		≥ 14 years	0.81
	Concern about aversive consequences	4–13 years	0.94
		≥ 14 years	0.82
NIAS	Picky eating	4–13 years	0.91
		≥ 14 years	0.80
	Fear	4–13 years	0.92
		≥ 14 years	0.89
	Low appetite	4–13 years	0.91
		≥ 14 years	0.85

Abbreviations: NIAS, nine item avoidant/restrictive food intake disorder screen; PARDI‐AR‐Q, Pica, ARFID and Rumination Disorder Interview‐ARFID‐Questionnaire.

### 3.3 ARFID Psychopathology (PARDI‐AR‐Q and NIAS Scores) Over the Two Age Samples

Table 3 shows the (subscale) scores of both samples on the PARDI‐AR‐Q and the NIAS for males and females who were not exhibiting symptoms of an eating disorder driven by overvaluation of shape and weight (EDE‐Q global‐score < 2.93). Only the PARDI‐AR‐Q “fear of aversive consequences” subscale scores differed over the two age samples (*t* (298) = 0.7, *p* = 0.035), suggesting that the severity of this profile was higher in the sample aged 4–13 years. In addition, the NIAS “picky eating” (*t* (298) = 1.3, *p* = 0.037) was higher, and “fear” (*t* (298) = −0.1, *p* = 0.027) scores were lower in the sample aged 4–13 years.

**TABLE 3:** Mean NIAS and PARDI‐AR‐Q subscale scores, and the proportion of participants who scored above the NIAS clinical cutoffs among individuals who did not exhibit symptoms of an eating disorder driven by overvaluation of shape and weight.

Self report	Subscale	Age category	Sex					
			Males	Range	Above cutoff, *N*, %	Females	Range	Above cutoff, *N*, %
			Subscale scores, mean (SD)			Subscale scores, mean (SD)		
NIAS	Picky eating	4–13 years	5.5 (4.8)	0.0–10.0	4/16, 25%	9.7 (4.0)	6.0–14	6/18, 33.3%
		≥ 14 years	3.6 (3.2)	0.0–11.0	9/114, 7.5%	5.3 (4.1)	0.0–15.0	25/150, 16.7%
	Fear	4–13 years	2.5 (1.3)	0.0–5.0	0/16, 0.0%	1.5 (1.3)	0.0–3.0	0/18, 0.0%
		≥ 14 years	2.7 (1.9)	0.0–9.0	6/114, 5.2%	3.8 (3.6)	0.0–15.0	22/150, 14.7%
	Low appetite	4–13 years	3.2 (2.8)	0.0–6.0	0/16, 0.0%	2.1 (1.7)	0.0–4.0	0/18, 0.0%
		≥ 14 years	4.0 (2.8)	0.0–15.0	6/114, 5.2%	4.3 (3.6)	0.0–15.0	12/150, 8.0%
	Total score	4–13 years	10.3 (8.8)	0.0–20.0	NA	12.7 (5.1)	7.0–17.0	NA
		≥ 14 years	8.5 (7.9)	0.0–29.0	NA	13.2 (9.5)	0.0–44.0	NA
PARDI‐AR‐Q	Avoidance based on sensory characteristics	4–13 years	0.9 (0.8)	0.0–1.7	NA	2.9 (2.4)	0.0–5.7	NA
		≥ 14 years	0.5 (1.0)	0.0–3.7	NA	1.2 (0.7)	0.0–5.3	NA
	Fear of aversive consequences	4–13 years	0.0 (0.0)	0.0–0.0	NA	1.3 (1.2)	0.3–2.7	NA
		≥ 14 years	0.9 (0.4)	0.0–3.0	NA	1.2 (0.7)	0.0–6.0	NA
	Lack of interest	4–13 years	0.8 (0.5)	0.0–1.7	NA	1.3 (0.6)	1.0–2.0	NA
		≥ 14 years	1.0 (1.0)	0.0–4.7	NA	1.6 (1.4)	0.0–6.0	NA
	Severity of impact	4–13 years	1.3 (0.6)	0.0–5.0	NA	2.4 (1.8)	0.0–4.5	NA
		≥ 14 years	1.0 (1.6)	0.0–3.5	NA	1.3 (0.8)	0.0–6.0	NA
	Diagnostic prediction	4–13 years	NA	NA	0/16, 0.0%	NA	NA	0/18, 0.0%
		≥ 14 years	NA	NA	2/114, 1.8%	NA	NA	60/150, 40.0%

Abbreviations: NIAS, nine item avoidant/restrictive food intake disorder screen; PARDI‐AR‐Q, Pica, ARFID and Rumination Disorder Interview‐ARFID‐Questionnaire.

### 3.4 Estimated Prevalence of ARFID Symptoms

Table 3 shows the prevalence of males and females exhibiting ARFID symptoms and ARFID phenotypes.

#### 3.4.1 NIAS, Sample Aged 4–13 Years

Addressing the second aim of estimating the prevalence of ARFID symptoms, including the prevalence of the three ARFID phenotypes, a total of 10/34 (29.4%) exceeded the clinical cutoff for picky eating and exhibited therefore ARFID symptoms. None of the participants in this age group exceeded the cutoff on the fear and low appetite subscales.

#### 3.4.2 NIAS, Sample Aged ≥ 14 Years

Supporting the second aim by identifying sensory‐based avoidance as the most common phenotype in both age groups, a total of 34/264 (12.8%) of the sample aged ≥ 14 years exceeded the clinical cutoff on picky eating. For the fear subscale, this was 28/264 (10.6%), and for the low appetite subscale, it was 18/264 (6.8%).

#### 3.4.3 PARDI‐AR‐Q Diagnostic Prediction, Sample Aged 4–13 Years

Addressing the second aim of estimating the prevalence of ARFID symptoms, none of the participants scored a positive screen on the diagnostic prediction.

#### 3.4.4 PARDI‐AR‐Q Diagnostic Prediction, Sample Aged ≥ 14 Years

In accordance with the second aim of estimating the prevalence of ARFID symptoms, a total of 62/264 (23.4%) had a positive screen on the diagnostic prediction and therefore exhibited ARFID symptoms.

#### 3.4.5 Symptoms of an Eating Disorder Driven by Overvaluation of Shape and Weight

Table 1 shows that a large proportion of the participants exhibited symptoms of an eating disorder driven by overvaluation of shape and weight, and an independent samples *t*‐test showed that the EDE‐Q global scores did not differ across the two age‐related samples. In the full sample, based on PARDI‐AR‐Q diagnostic prediction and an EDE‐Q global score above the clinical cutoff, 22/433 (5.1%) of participants aged ≥ 14 years exhibited both symptoms of ARFID and of an eating disorder driven by overvaluation of shape and weight, a comorbidity not observed in the 4–13 years age group.

### 3.5 Correlates

#### 3.5.1 Sample Aged 4–13 Years

Addressing the third aim, the sample size was too small to conduct a regression analysis with the PARDI‐AR‐Q severity of impact score, with the NIAS total score as the dependent variable.

#### 3.5.2 Sample Aged ≥ 14 Years

In accordance with the third aim, only the EDE‐Q global score (*B* = 0.8; *r* = 0.5; *p* < 0.001; moderate effect) was positively associated with the PARDI‐AR‐Q severity of impact score. With regard to the NIAS total score, the EDE‐Q global score (*B* = 5.2; part *r* = 0.4; *p* < 0.001; moderate effect) and female sex (*B* = 3.9; part *r* = 0.2; *p* = 0.008; small effect) were positively associated. Age was negatively associated with the NIAS total score (*B* = −0.1; part *r* = −0.1; *p* = 0.023; small effect). BMI and comorbid psychopathology did not contribute to the model and were therefore excluded.

## 4 Discussion

The aim of the present study was to explore concurrent validity between the PARDI‐AR‐Q and the NIAS, to estimate the prevalence of ARFID symptoms, including those of the three phenotypic expressions, and to identify potential correlates of ARFID psychopathology in the GCC among children (4–13 years) and adolescents/adults (≥ 14 years), excluding individuals with eating disorder symptoms driven by the overvaluation of shape or weight. Concurrent validity between the PARDI‐AR‐Q and the NIAS was supported. Based on the PARDI‐AR‐Q, the findings suggest that approximately 23% of individuals aged ≥ 14 exhibited ARFID symptoms. Consistent with both clinical observations and Western studies (Zimmerman and Fisher 2017), the NIAS indicated that sensory‐based food avoidance was the most commonly reported symptom, with 29.4% of children (4–13 years) and 12.8% of adolescents/adults (≥ 14 years) exceeded the clinical cutoff in the GCC. Notably, none of the children aged 4–13 years exceeded the clinical cutoff on the subscales concerned about aversive consequences or lack of interest in food. Several factors may explain why concerns about aversive consequences and lack of interest in food did not exceed the cutoff in this age group. These symptoms may be less pronounced due to developmental stages, where cognitive and emotional reflection is still developing, or because parents may have difficulty accurately assessing these phenotypes compared to the child's own self‐report (Watts et al. 2023).

Socio‐cultural factors, dietary norms, and food availability influence food preferences and consumption behaviors (Hiamey et al. 2021). Globalization shifted traditional dietary practices towards Western foods (Melisse et al. 2024a), designed to maximize sensory appeal, being high in salt, sugar, and fat (Fazzino et al. 2019). In addition, the oil boom resulted in increased affluence. As a result, families tend to rely less on home‐cooked meals, resulting in a decrease in their appeal (Shrimpton and Rokx 2012). Consequently, individuals in the GCC tend to rely more on highly processed foods, which are more consistent in texture and flavor, leading to increased sensory sensitivity (Schatzker 2015), which warrants further socially situated research.

Prevalence estimates vary widely between children and adolescents (Sanchez‐Cerezo et al. 2023), but research suggested that the prevalence may diminish with age (Bertrand et al. 2021; Chen et al. 2019; Herle et al. 2020; Sanchez‐Cerezo et al. 2023). However, the results of the present study were inconclusive. Sensory‐based avoidance‐related symptoms (NIAS) were commonly reported in the group of 4–13 years old, but symptoms of fear of aversive consequences (NIAS), lack of appetite (NIAS), and ARFID diagnostic prediction (PARDI‐AR‐Q) were more commonly reported in the sample aged ≥ 14 years old. In addition, on both self‐reports, avoidance based on the sensory characteristics of food was more severe in the group aged 4–13 years old, indicating potential benefits of preventative programs for this age group. Furthermore, among the complete sample aged ≥ 14 years old, the reported severity of ARFID psychopathology reduced with age, which is consistent with prior studies (Bertrand et al. 2021; Chen et al. 2019; Herle et al. 2020; Sanchez‐Cerezo et al. 2023). Therefore, future studies implementing a third age class are recommended, including adolescents, to clarify ARFID symptom prevalence over various age groups and whether the severity decreases from adolescence to adulthood. Additionally, this would allow the more representative BMI standard deviation score to be used among this age group instead of BMI.

The present study found a positive association between ARFID psychopathology severity and eating disorder pathology driven by overvaluation of shape and weight. In addition, in the complete sample aged ≥ 14 years old, 5.1% of individuals exhibited symptoms for both types of eating disorder psychopathology. Despite eating disorders driven by overvaluation of shape and weight being an exclusion criterion for ARFID (APA 2022), comorbid pathology is frequently observed, with up to 14% of patients seeking treatment for eating disorders and 32% of those with feeding problems also meeting ARFID criteria (Hay et al. 2017; Williams et al. 2015). This overlap suggests that brief screening tools such as the NIAS (Zickgraf and Ellis 2018) and the 7‐item EDE‐Q (Fairburn and Beglin 2008) may be useful in clinical practice within the GCC. These tools could enhance the identification of ARFID symptoms in patients presenting with broader eating disorder symptoms, potentially facilitating earlier prevention, diagnosis, and treatment.

In the present study, females reported higher ARFID psychopathology severity. Interestingly, a larger proportion of females appeared to exhibit ARFID symptoms compared to males, which contrasts with prior research indicating that ARFID is more common in males (Cooney et al. 2018; Forman et al. 2014; Norris et al. 2014; Wong et al. 2022). The higher prevalence among females in the present study may reflect sample bias. Females in the GCC may have been more proactive in seeking information for eating‐related issues, potentially leading to a higher ARFID scores in the present study. This aligned with observations in clinical practice, as specialized eating disorder facilities reported the overrepresentation of females (Cooney et al. 2018; Forman et al. 2014; Norris et al. 2014). This association underscores the need for further research to explore sex differences in ARFID presentation, especially in non‐Western settings, like the GCC.

Other studies were inconclusive with regard to the role of BMI. Some studies found ARFID psychopathology to be associated with a low BMI (Strand et al. 2019), and others with a high BMI (Fonseca et al. 2024). The suggestions of the present study echo Kambanis et al. (2020) who found no association. Additionally, the present study failed to find an association between comorbid psychopathology and ARFID, which contradicts other studies that reported that ARFID was commonly associated with comorbid psychopathology (Archibald and Bryant‐Waugh 2023; Cooney et al. 2018; Kambanis et al. 2020; Sanchez‐Cerezo et al. 2023). It is possible that too few participants reported comorbid psychopathology, along with higher levels of ARFID psychopathology, potentially resulting in low power. On the other hand, the present study relied on self‐reported comorbid psychopathology, while investigator‐based assessments were more reliable. Future studies focusing on clinical samples using investigator‐based assessments are therefore recommended.

EDE‐Q global scores did not differ between the age samples (4–13 years and ≥ 14‐years). Since the COVID‐19 pandemic, there has been an increase in eating disorders, particularly driven by overvaluation of shape and weight among children between 9 and 13 years old (Spettigue et al. 2021). Additionally, the earlier onset of menarche, a significant precipitating factor for eating disorders, may contribute to the emergence of psychopathology in younger children at levels similar to those seen in adolescents (Biro and Kiess 2016).

While the present study offers valuable information, several limitations should be considered. The PARDI‐AR‐Q and NIAS instruments have not yet been validated within the GCC region, which may limit the cultural appropriateness and generalizability of the findings to this population. However, by using established and widely accepted scales, efforts were made to minimize reporting bias and enhance the validity of self‐reported data. Additionally, the reliance on self‐reports limited the ability to confirm clinical diagnoses of both eating disorder pathology and comorbid psychopathology. Furthermore, the cross‐sectional design precluded any causal inferences. Moreover, potential underrepresentation of participants from Bahrain, Kuwait, Oman, and Qatar may have skewed prevalence estimates, limiting generalizability to the entire GCC. Finally, the sample may have been biased toward individuals with an interest in mental health care, those with a higher level of education, and English‐speaking populations, which could further impact the representativeness of the findings. Future research should incorporate longitudinal designs, clinically validated assessment tools, and more diverse samples to refine ARFID prevalence estimates and explore age‐related changes in severity within the GCC context.

Future studies should also consider using investigator‐measured BMI, as self‐reported weight and height are subject to bias (Gorber et al. 2007) and use more relevant age‐related groups to examine the association with BMI Standard Deviation Score. The sample of participants aged 4–13 years was sex‐balanced. However, the sample aged ≥ 14 showed some sex imbalances (WorldBank 2024), though it was more balanced than previous studies (AlShebali et al. 2021; Melisse et al. 2022a). Considering that 70% of participants were between the ages of 14–40, the findings predominantly apply to individuals under 40, which may limit their generalizability. Moreover, it is broadly reflective of the GCC population with approximately 37% under the age of 18% and 70% under the age of 40 (WorldBank 2024). The present study is the first study to estimate the prevalence of ARFID symptoms in the GCC, underscoring the need for larger, more inclusive, and culturally representative research in the region. The PARDI‐AR‐Q used in the study has not yet been validated in the GCC, and while the NIAS was validated in Lebanon, cultural differences may affect its applicability in other Arab countries. As recommended for this context, we are currently collecting data in a clinical sample and thus are in the process of validating these instruments. Finally, public health initiatives are recommended to integrate ARFID‐management strategies to address this emerging challenge, potentially reducing the long‐term physical and psychosocial impacts associated with the disorder. Overall, this study tentatively proposes directions for future research and underscores the potential value and importance of culturally adapted approaches in the assessment and treatment of ARFID in the GCC.

## Author Contributions

**Bernou Melisse:** conceptualization, data curation, formal analysis, investigation, methodology, project administration, writing – original draft. **Hassan Fakhri:** data curation, methodology, writing – review and editing. **Lynne Kennedy:** data curation, writing – review and editing. **Maria J. Figueiras:** data curation, writing – review and editing. **Munirah Alshebali:** resources. **Hala Abu Taha:** data curation. **Carine el Khazen:** data curation, writing – review and editing. **Dalal Alkazemi:** writing – review and editing. **Sandra Mulkens:** supervision, writing – review and editing.

## Ethics Statement

Ethical approval was obtained on 11‐07‐2024 by the ethical board of the American Center for Psychiatry and Neurology (reference number ACPN_0062).

## Consent

Informed consent was obtained from all participants included in the present study.

## Conflicts of Interest

The authors declare no conflicts of interest.

## Data Availability

Upon reasonable request with BM.
